# Supplementary material for: Vaccination to prevent T cell subversion can protect against persistent hepacivirus infection
Source: Nat Commun. 2019 Mar 7;10:1113. doi: 10.1038/s41467-019-09105-0 (PMC6405742; doi:10.1038/s41467-019-09105-0)
Supplement: Supplementary file 1 — Supplementary Information [file 41467_2019_9105_MOESM1_ESM.pdf]

Supplementary Materials for

Vaccination to prevent T cell subversion can protect against persistent hepacivirus infection

Hartlage, et al. 2019

PDF file includes (in order of appearance in text):

Supplemental Figure 1. Gating strategy for quantification of intracellular cytokines.

Supplemental Table 1. Core-specific MHC class II epitopes.

Supplemental Table 2. List of PCR primers used in study.

Supplemental Figure 2. Gating strategies for analysis of tetramer positive cells.

Supplemental Figure 3. Absence of in vivo killing during acute RHV infection.

Supplemental Figure 4. Immunogenicity of Ad-NSmut and characterization of vaccine-elicited T cell repertoire.

Supplemental Figure 5. Serum ALT values in vaccinated rats.

Supplemental Figure 6. Mapping of $NS3_{974}$ class I epitope.

Supplemental Figure 7. RHV-specific T cell response in Ad-null vaccinated rats after virus challenge.

Supplemental Figure 8. RHV-specific $CD4^+$ T cell responses in depleted rats after virus challenge.

Supplemental Figure 9. Class I epitope evolution in CD4-depleted rats.

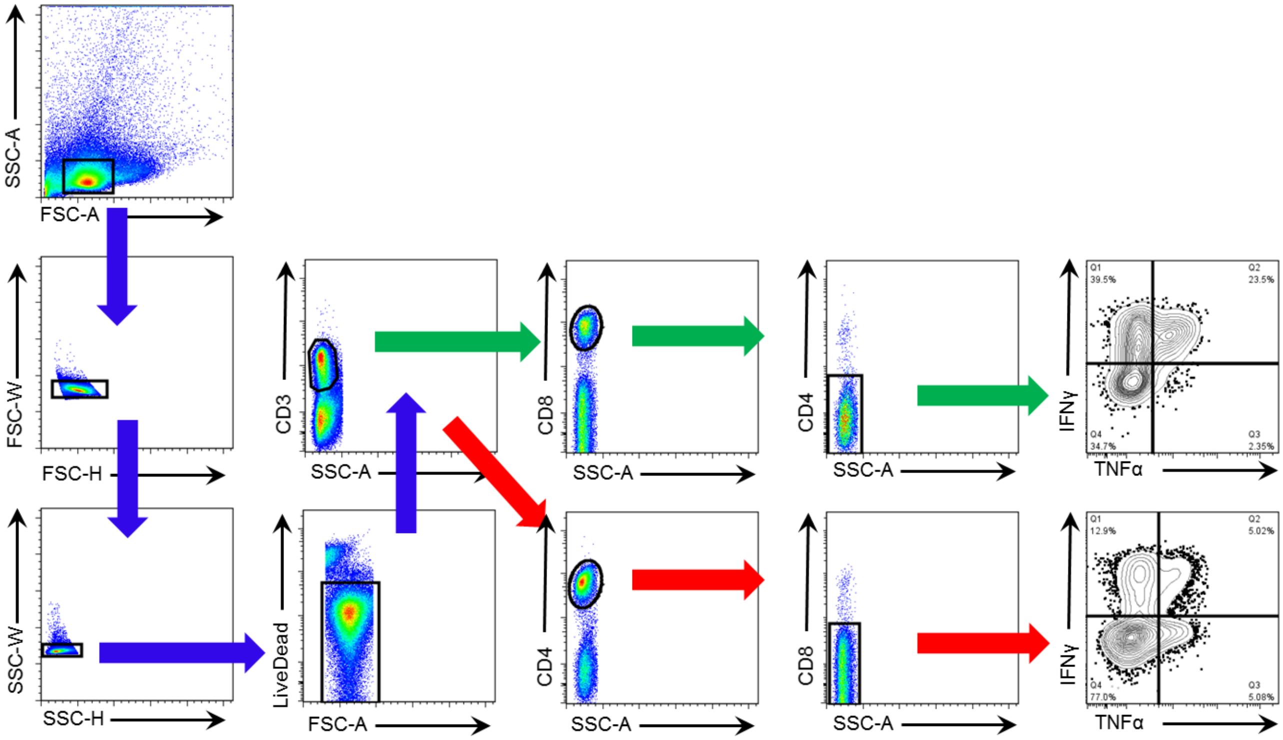

Supplemental Figure 1. Gating strategy for quantification of intracellular cytokines. Shown are intrahepatic mononuclear cells after 5-hr PMA/Ionomycin stimulation in the presence of brefeldin A.

Supplemental Table 1. Core-specific MHC class II epitopes.

Peptide	Position	Sequence
P9	$Core_{57}$	AKRRRRHRRDQGGWRRSP
P12	$Core_{78}$	VDPYVRQGLQILLPSAAY
P13	$Core_{85}$	GLQILLPSAAYPVRDPRR
P17	$Core_{113}$	GTLGWTADLLHHVPLVGP
P18	$Core_{120}$	DLLHHVPLVGPLVGHPAR
P20	$Core_{134}$	HPARLICRAVRACEDGIN
P22	$Core_{148}$	DGINSFTGIAGVHLFLIC

Supplemental Table 2. List of PCR primers used in study.

Name	Sequence (5'→3')
GNN-F1	GATTTGTGGAAATAACACGGTGTGCATATTTG
GNN-R1	AACCAGCTGGGTGACGCG
5'KpnI_NS35B_F	GATGGGGTACCATGGCCCCCTTCAACTTAAG
3'EcoRV_NS35B_R	GGTGGTAAGATATCGAGGTTGAGGGATTTACC
RHV_NS3amino-F	CCGCTGAGGACACTGGTTTCA
RHV_NS3amino-R	CGGCATGGCACTCGTCGC
RHV_NS3carboxy-F	CCATGATAACGGCTCAAGGCTC
RHV_NS3carboxy-R	GTCGCCCGTCAAGTCCCAGG
RHV_NS3amino-F2	CCGCTGAGGACACTGGTTTCA
RHV_NS3amino-R2	CGTGGCCGTCGCAAGAAGC
RHV_NS4B-F	GCTTCCAGCATCCAGGGGTG
RHV_NS4B-R	CGGGTCGGCAGGCGTAGAGT
RHV_NS5A-F	CAAGCCACCACACTCAGACAAAC
RHV_NS5A-R	TGGGATTACGCATCAAGCCG
RHV_NS5Bamino-F	GCCCACAGACCCAGACACTTTCA
RHV_NS5Bamino-R	GCTTCATGGCCACTGCGAAT
RHV_NS5Bcarboxy-F	TTACATGACAACCTGTATGCTGG
RHV_NS5Bcarboxy-R	ACATCAGGAGAAGATGAAGGCTAT
NS3aseq-F1	CCCTAAAGGGCTTACCAGTG
NS3aseq-F2	TCTTTCTAAGGTGCAGGAGC
NS3cseq-F1	CATGTTCCTAGCGAAGAACC
NS3cseq-F2	TGATGCCAACATTCAAGCTG
NS4Bseq-F1	GAAGAATACTTTGCTGAGACGG
NS4Bseq-F2	CTACAAAGCTAACAACACAGACG
NS5Aseq-F1	AAGTGGCTGGAAACAGCCTC
NS5Aseq-F2	CTGTAGGCTGGACTAGTATGGC
NS5Aseq-F3	TGCACACATGAAAGACATCTCACG
NS5Aseq-F4	GTGCATCCTGTTCCAGAATC
NS5Baseq-F1	AGTCGTCTGGATCATGGACC
NS5Baseq-F2	TGGTGGAGAAGATGGTGCTC
NS5Bcseq-F1	CATGGTGATGCAAGGTGCTG
NS5Bcseq-F2	GTGTGTGCAATTCTTACAGCAG

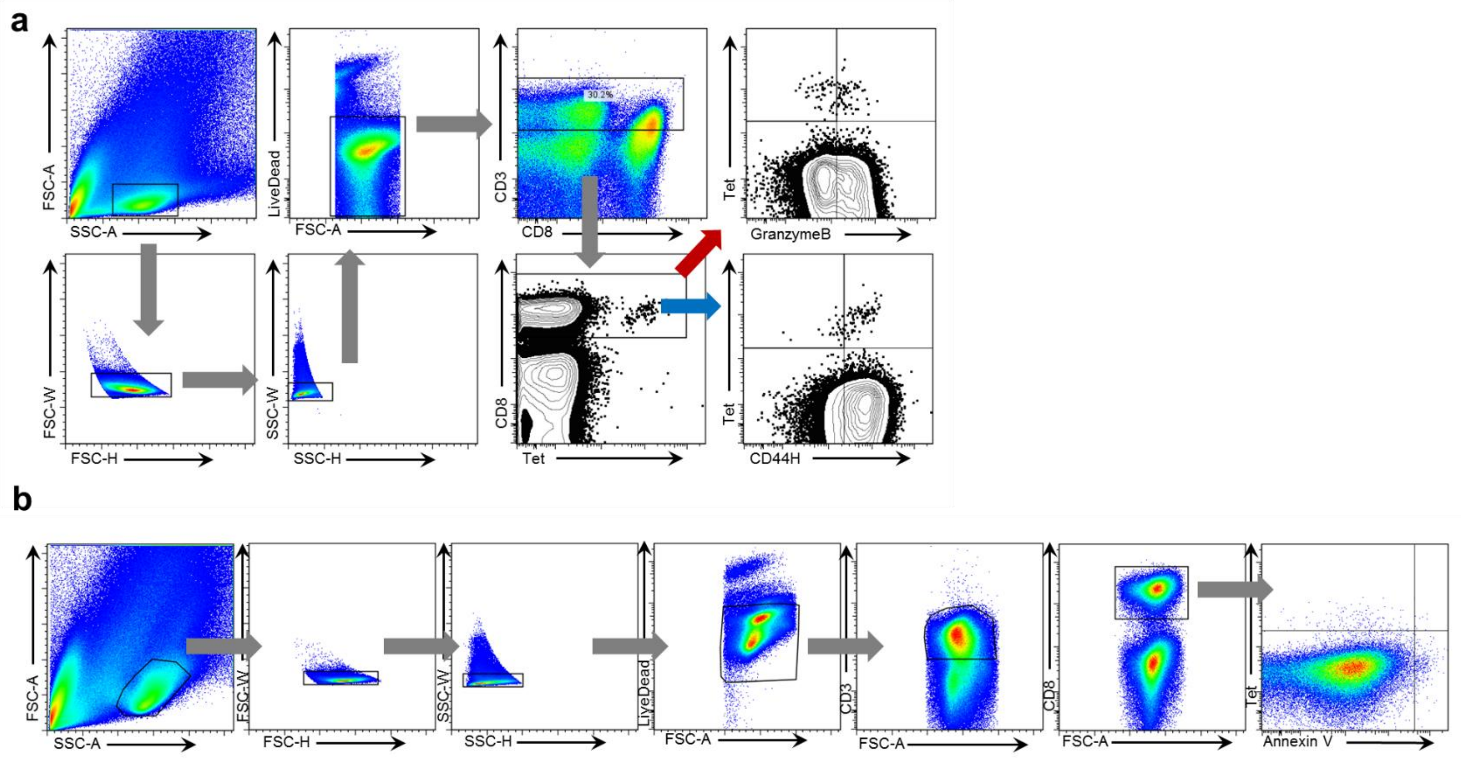

Supplemental Figure 2. Gating strategies for analysis of tetramer positive cells. Analysis of tetramer positive cells after staining with markers for CD44H and granzyme B (a) or Annexin V (b).

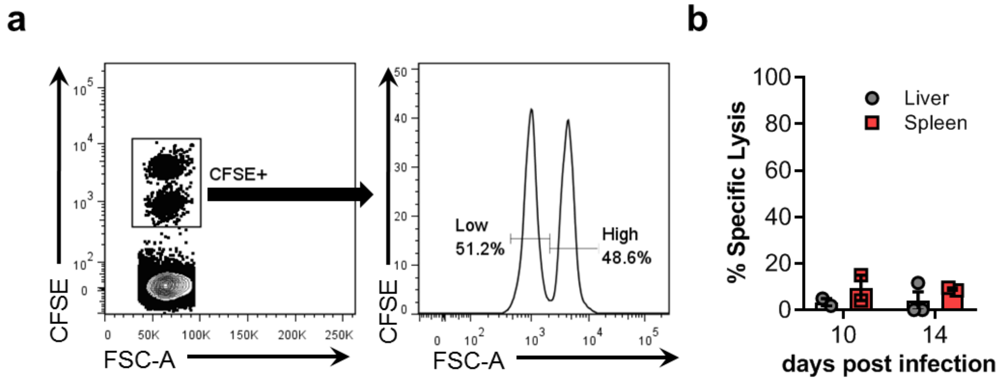

Supplemental Figure 3. Absence of in vivo killing during acute RHV infection. (a) Representative FACS plots of $CFSE^{+}$ cells in infected liver at 10 days post infection. **(b)** Summary of percent specific lysis of RHV-rn1 peptide-loaded cells at days (d) 10 and 14 post infection in livers (grey circles) and spleens (red squares).

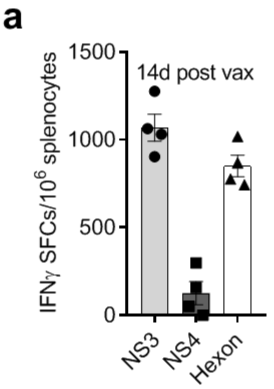

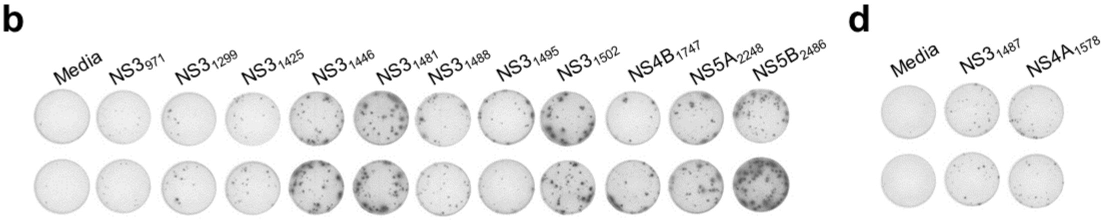

c

MHC class II epitopes

Position	Sequence
$NS3_{971}$	YNGSICVIGTPLSRFMGF
$NS3_{1299}$	IQKGRHLIFQTSKSHCDN
$NS3_{1425}$	IVPDACIYEAFDSGLAYF
$NS3_{1446}$	PAEVATHLSFYHNQVGLP
$NS3_{1481}$	YVQSNYLEMMKNRVDSYT
$NS3_{1488}$	EMMKNRVDSYTYLYAAQY
$NS3_{1495}$	DSYTYLYAAQYQLAKAEG
$NS3_{1502}$	AAQYQLAKAEGAMAPNDN
$NS4B_{1747}$	MVGHAFLTYGSATSACLV
$NS5A_{2248}$	MELLREYETSNDHVPKED
$NS5B_{2486}$	SGKTEIVKTLYSKLEEGI

e

MHC class I epitopes

Position	Sequence
$NS3_{1497}$	YTYLYAAQY
$NS4A_{1578}$	CVFMAIDLF

Supplemental Figure 4. Immunogenicity of Ad-NSmut and characterization of vaccine-elicited T cell repertoire. Lewis rats were vaccinated intramuscularly with 5 x 10^8 ifu Ad-NSmut. Two weeks after vaccination, splenocytes were harvested for analysis of cellular immunity. **(a)** IFNγ ELISpot responses against vaccine insert (NS3, NS4; 2 µg/mL) and backbone (Hexon; 0.6 nmol/mL) peptides. Data from n=3 rats per group is shown (mean ± SEM). **(b-e)** Splenocytes from four rats were pooled together at equal ratios, magnetically enriched for $CD8^{pos}$ and $CD8^{neg}$ cells, and used to map MHC class I and II epitopes in the NS3-5B insert by IFNγ ELISpot assay. MHC class II epitopes (b and c) were identified by testing individual NS3-4B peptides and NS5B matrix pools. MHC class I epitopes (d and e) for the RT1-A^{l} protein of the Lewis rat were screened and confirmed using the SYFPEITHI prediction algorithm. Panels b and d show representative ELISpot responses of identified epitopes in duplicate.

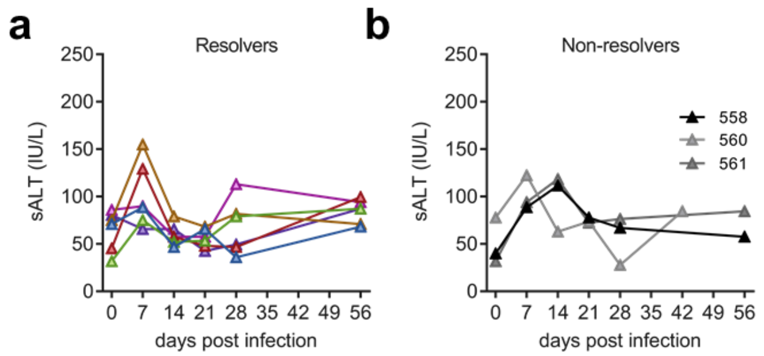

Supplemental Fig 5. Serum ALT values in vaccinated rats. Levels of serum ALT (sALT) in (a) resolvers and (b) non-resolvers after RHV infection.

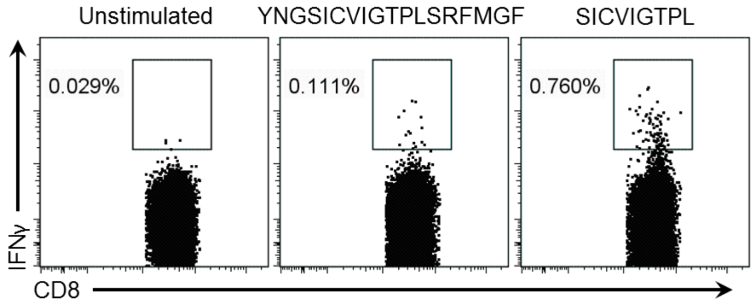

Supplemental Fig. 6. Mapping of NS3$_{974}$ class I epitope. Flow cytometric analysis of intracellular IFNγ production by CD8$^+$ T cells in immune splenocytes following 5-hr stimulation with untruncated or truncated NS3$_{974}$ peptide.

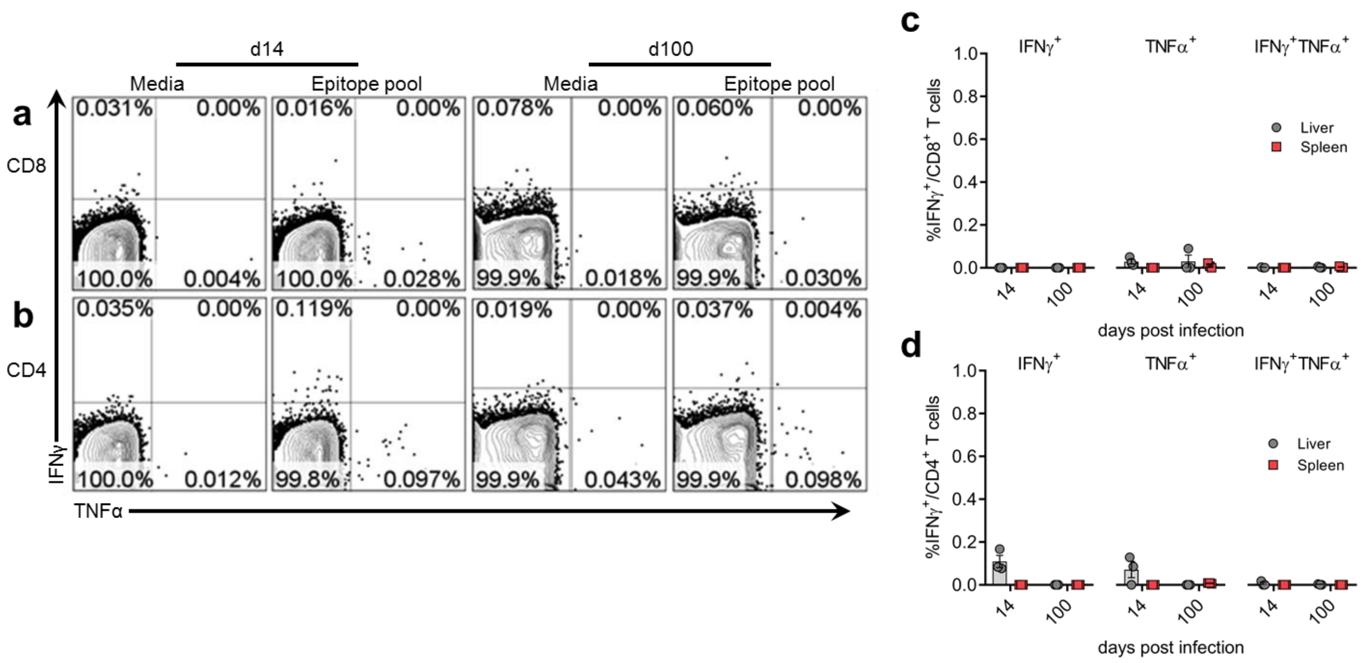

Extended Figure 7. RHV-specific T cell response in Ad-null vaccinated rats after virus challenge. Lewis rats were vaccinated intramuscularly with 5 x 10^8 ifu Ad-null. Three weeks after vaccination, rats were challenged intravenously with 10^6 genomes RHV. At days 14 and 100 post infection, virus-specific T cell responses were quantified in spleens and livers by flow cytometric analysis following 5-hr stimulation with a pool of MHC class I and II epitopes (5 µg/mL). **(a,b)** Representative flow plots of intracellular IFNγ and TNFα cytokine production in $CD8^+$ (a) and $CD4^+$ (B) T cells from livers of vaccinated rats. **(c,d)** Calculated frequencies of total and dual positive cytokine producers in $CD8^+$ (c) and $CD4^+$ (d) T cell compartments following RHV-specific peptide stimulation. Liver, grey circles; spleen, red squares. Data from n=3 rats per group are shown (mean ±SEM).

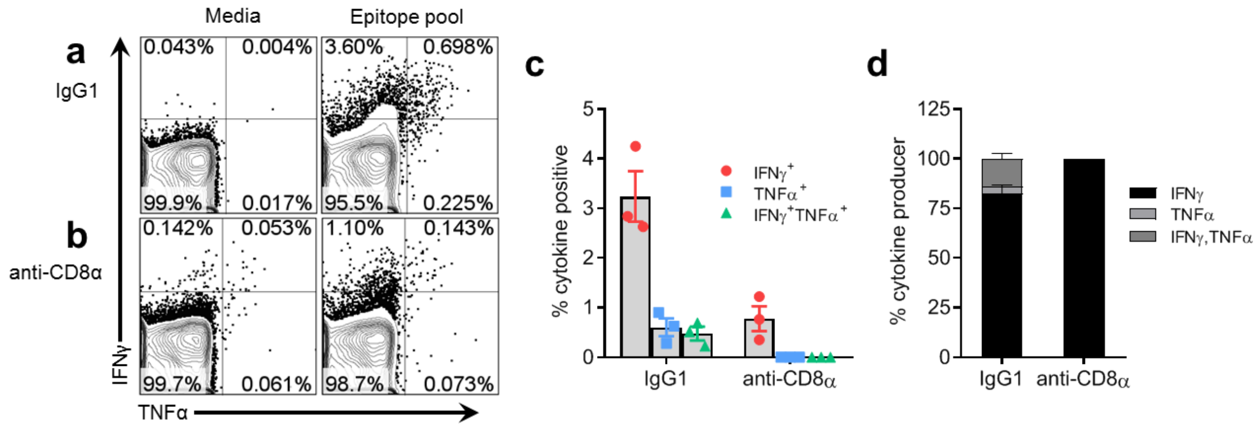

Supplemental Figure 8. RHV-specific CD4$^+$ T cell responses in depleted rats after virus challenge. Lewis rats were vaccinated intramuscularly with 5 x 10^8 ifu Ad-NSmut. Three weeks after vaccination, rats were challenged intravenously with 10^6 genomes RHV. At -2 and 5 days post infection, rats were depleted of CD8α$^+$ cells by antibody or isotype control. At day 9 post infection, CD4$^+$ T cell responses were quantified in liver by flow cytometric analysis following 5-hr stimulation with a pool of MHC class I and II epitopes (5 µg/mL). **(a,b)** Representative flow plots of intracellular IFNγ and TNFα cytokine production by intrahepatic CD4$^+$ T cells in (a) mock and (b) CD8α$^+$ cell-depleted rats. **(c)** Calculated frequencies of total and dual positive cytokine producing cells in mock and CD8α$^+$ cell-depleted rats. IFNγ, red circles; TNFα, blue squares; IFNγ,TNFα, green triangles. Data from n=3 rats per group are shown (mean ±SEM). **(d)** Relative frequency of intrahepatic CD4$^+$ T cells producing dual or single IFNγ or TNFα cytokines. Data from n=3 rats per group are shown (mean ±SEM).

day 21	$NS3_{974}$		$NS3_{1497}$		$NS4A_{1578}$
	S I C V I G T P L		Y T Y L Y A A Q Y		C V F M A I D L F
506	- - - - - - - - -	506	- - - - - - - - -	506	- - - - - - - - -
507	- - - - - - - - -	507	- - - - - - - - -	507	- - - - - - - - -
508	- - - I - - - - -	508	- - - - - - - - -	508	- - - - - - - - -
509	- - - - - - - - -	509	- - - - - - - - -	509	- - - - - - - - -

Supplemental Figure 9. Class I epitope evolution in CD4-depleted rats. Serum viral RNA in CD4-depleted rats at 21 days post infection was analyzed for presence of mutations in class I-restricted epitopes by consensus PCR sequencing. A single non-synonymous V→I mutation was identified at position 4 in the $NS3_{974}$ epitope of serum virus recovered from rat 508.
